# Supplementary material for: Chromothripsis during telomere crisis is independent of NHEJ, and consistent with a replicative origin
Source: Genome Res. 2019 May;29(5):737–49. doi: 10.1101/gr.240705.118 (PMC6499312; doi:10.1101/gr.240705.118)
Supplement: Supplemental Material [file supp_gr.240705.118_Supplemental_file_1.zip › contigs/annotated_contigs/DB71/contig.2.DB71_length_378_mean_cov_4.81216931217.docx]

**DB71_length_378_mean_cov_4.81216931217**

```
TTCTTGCCTTCCTTCTGCACAATGTTTACAAATTCAGAACATATGCTGAAACACTCCAAATGAACTTGACTTCTGCTGCTTCTCTTTCT
 >chr3:61446491-61446654 + E=1e-83
TTGGGAAACCACCTCATTTTTTGTGGACTAGCTGGCCTCCCCTCTCATCTTGACGCCTTTCTCCAGTTGTGATT|TCT|CCAACCTAGT
                                                                              >chr3:609
GAACAATGCCAGTCTGGGACCCAAGGACTATTTACTCAACAGAAGAATTCCAACTTCAGGCAGGGGATAGGCGAAGTCCAGATAACATC
33415-60933627 + E=3e-116
TAGATATTTCTTGACATTGCATTTGATTGGAAATAAAGGAAAAGTAAAGCATTTCTGGTTAGTTTCTGTTTCTTAGTGTCTCTAGTTCC

CATTTTATTTCAATCAAAATATCA
```
